# The Independent Practitioner

**Published:** 1882-12

**Authors:** 


*The Independent Practitioner.* We learn that W. C. Barrett. M. D., D. D. S., of Buffalo, N. Y. has accepted the entire charge of the dental department of this publication ; and that all den-tal matter is to be sent to him. We congratulate the proprietor of the Practitioner in securing the services of so able an editor, and predict for it increasing prosperity, interest and usefulness.

